# Effects of Aging and Idiopathic Parkinson’s Disease on Tactile Temporal Order Judgment

**DOI:** 10.1371/journal.pone.0118331

**Published:** 2015-03-11

**Authors:** Natsuko Nishikawa, Yasushi Shimo, Makoto Wada, Nobutaka Hattori, Shigeru Kitazawa

**Affiliations:** 1 Department of Neurology, Juntendo University School of Medicine, Tokyo, 113-8421, Japan; 2 Department of Neurophysiology, Graduate School of Medicine, Juntendo University, Tokyo, Japan; 3 Developmental Disorders Section, Department of Rehabilitation for Brain Functions, Research Institute of National Rehabilitation Center for Persons with Disabilities, Tokorozawa, Japan; 4 Department of Brain Physiology, Graduate School of Medicine, Osaka University, Osaka, Japan; 5 Dynamic Brain Network Laboratory, Graduate School of Frontiers Bioscience, Osaka University, Osaka, Japan; University of Reading, UNITED KINGDOM

## Abstract

It is generally accepted that the basal ganglia play an important role in interval timing that requires the measurement of temporal durations. By contrast, it remains controversial whether the basal ganglia play an essential role in temporal order judgment (TOJ) of successive stimuli, a behavior that does not necessarily require the measurement of durations in time. To address this issue, we compared the effects of idiopathic Parkinson’s disease (PD) on the TOJ of two successive taps delivered to each hand, with the arms uncrossed in one condition and crossed in another. In addition to age-matched elderly participants without PD (non-PD), we examined young healthy participants so that the effect of aging could serve as a control for evaluating the effects of PD. There was no significant difference between PD and non-PD participants in any parameter of TOJ under either arm posture, although reaction time was significantly longer in PD compared with non-PD participants. By contrast, the effect of aging was apparent in both conditions. With their arms uncrossed, the temporal resolution (the interstimulus interval that yielded 84% correct responses) in elderly participants was significantly worse compared with young participants. With their arms crossed, elderly participants made more errors at longer intervals (~1 s) than young participants, although both age groups showed similar judgment reversal at moderately short intervals (~200 ms). These results indicate that the basal ganglia and dopaminergic systems do not play essential roles in tactile TOJ involving both hands and that the effect of aging on TOJ is mostly independent of the dopaminergic systems.

## Introduction

Patients with Parkinson’s disease (PD), a neurodegenerative disease strongly associated with basal ganglia dysfunction, show deficits in temporal processing when performing temporal reproduction tasks [1–3], perceptual timing tasks [4, 5], simultaneity judgments, and temporal discrimination between two stimuli [6–8]. A neuroimaging study of healthy participants confirmed that the basal ganglia and the substantia nigra pars compacta are involved in the reproduction of short and long time intervals [9]. These previous findings suggest that dopaminergic transmission in the basal ganglia and related cortical structures play an important role in interval timing tasks [10, 11].

In contrast to the general agreement regarding the contribution of the basal ganglia to interval timing, it remains controversial whether the basal ganglia play an essential role in judging the temporal order of two successive tactile stimuli. When participants were required to judge the temporal order of two successive tactile stimuli, delivered to different fingers on the same hand, Fiorio, Valente, Gambarin, Bentivoglio, Ialongo, Albanese, Barone, Pellecchia, Brancati, Moretto, Fiaschi and Tinazzi [7] reported that the threshold interval to judge the order of the two stimuli was significantly larger in participants with familial PD due to a PINK1 mutation compared with age-matched healthy participants. By contrast, Nelson, Premji, Rai, Hoque, Tommerdahl and Chen [12] reported that the threshold interval in patients with idiopathic PD was similar to age-matched controls, even during periods without medication. This discrepancy may be due to differences in the genetic backgrounds of the two patient groups, but it is worth noting that Fiorio, Valente, Gambarin, Bentivoglio, Ialongo, Albanese, Barone, Pellecchia, Brancati, Moretto, Fiaschi and Tinazzi [7] required the participants to judge both simultaneity and temporal order, whereas Nelson, Premji, Rai, Hoque, Tommerdahl and Chen [12] required the participants to only judge temporal order. Because the information processing required for temporal order judgment (TOJ) is proposed to be different from that required for simultaneity judgment (SJ) [13–15], and the two processes have been modeled to work in parallel [16], task performance in TOJ should have been disturbed by the concurrent SJ task in the study by Fiorio, Valente, Gambarin, Bentivoglio, Ialongo, Albanese, Barone, Pellecchia, Brancati, Moretto, Fiaschi and Tinazzi [7]. Assuming that PD patients showed impaired performance in the SJ task, the TOJ task must have been more disturbed by the concurrent SJ task in PD patients than in age-matched controls.

In addition, a recent neuroimaging study examining the neural correlates of tactile TOJ suggested major contributions from several cortical areas (the prefrontal, parietal, and temporal cortices) but less contributions from the basal ganglia [17]. Thus, it is possible that the basal ganglia are not critically involved in TOJ. The primary purpose of the present study was to test whether the performance of tactile TOJ in idiopathic PD patients is significantly different from age-matched controls.

In contrast to previous studies [7, 12] in which two stimuli were delivered to the fingers of one hand, we used a TOJ task whereby two successive tactile stimuli were delivered to each hand. Involving both hands in our TOJ task is unique because crossing the arms causes a reversal of subjective temporal order in healthy young participants at intervals of up to 300 ms [13, 18]. Thus, to examine whether the basal ganglia contribute to the reversal of subjective temporal order due to arm crossing, we compared TOJ in PD and non-PD participants not only in the standard arms-uncrossed condition but also in the arms-crossed condition. We also studied young healthy participants as another control group. Because young PD patients are rare, it was not possible to adopt a factorial design (PD/non-PD x young/old), but we expected that the effect of aging [19–24] could serve as a control for evaluating the effects of PD. Therefore, we examined the TOJ of tactile stimuli delivered to each hand with arms uncrossed in one condition and crossed in another in young and elderly participants without PD and in idiopathic PD patients.

## Methods

### Participants

Twenty young adults (9 females and 11 males; mean age 23 ± 1.0 years; mean ± standard deviation), 26 patients with idiopathic Parkinson’s disease (PD patients; 12 females and 14 males; mean age 67 ± 4.9 years), and 24 age-matched elderly adults without PD (non-PD elderly participants; 12 females and 12 males; mean age 67 ± 4.9 years) participated in this study (Table 1). All of the participants were strongly right-handed (+60 ≤ L.Q ≤ +100) according to the Edinburgh Inventory [25]. The diagnosis of PD was based on the United Kingdom Parkinson’s Disease Society Brain Bank Clinical Diagnostic Criteria [26]. Disease severity was assessed with the Unified Parkinson’s Disease Rating Scale (UPDRS) Part III and the Hoehn-Yahr Scale: H&Y [27]. All of the PD patients were receiving dopaminergic replacement therapy, which included levodopa (0–1000 mg) and/or a dopamine agonist (pramipexole, ropinirole, or pergolide), or entacapone or selegiline. The PD patients joined the experiments during periods under medication. The UPDRS-III was performed when patients were on (mean ± S.E. UPDRS-III-on = 13.8 ± 6.7) and off (UPDRS-III-off = 30.6 ± 8.8) their medication. The Hoen-Yahr stage when patients were off medication ranged from mild to advanced (minimum, 1; maximum, 4). None of the patients had a history of stroke, head injury, epilepsy, psychiatric problems, any primary sensory abnormalities or diabetes mellitus. The Mini-Mental State Examination (MMSE) [28] was performed as a brief mental screening test for the PD patients and non-PD elderly participants. All of the participants scored 25 or more (full score = 30) except for one PD patient. During the experiments, one PD and another non-PD elderly participant were not able to keep their eyes closed as instructed. These two participants and the PD patient with the MMSE score less than 25 were excluded from further analysis. Therefore, we analyzed data from 20 young participants, 23 non-PD elderly participants, and 24 PD participants. Written informed consent was obtained from all participants prior to the experiments. This study was approved by the Ethical Review Board of Juntendo University School of Medicine.

**Table 1:** Demographics of the participants.

	Young	Elderly	PD
Number	20	23	24
Gender (M: F)	9:11	11:12	14:10
Age (years)	23 ± 1.0	67 ± 4.9	67 ± 4.9
Edinburgh handedness inventory	89 ± 13	92 ± 15	94 ± 11
Mini Mental State Examination	NA	29 ± 1.7	28 ± 1.6
Self-Rating Depression Scale *	NA	27 ± 4.0	41 ± 9.0
Disease duration (years)	NA	NA	8.7 ± 4.4
UPDRS III total motor score, On	NA	NA	13 ± 6.7
UPDRS III total motor score, Off	NA	NA	32 ± 9.7
Hoen-Yahr stage, On	NA	NA	2.4 ± 0.60
Hoen-Yahr stage, Off	NA	NA	3.7 ± 0.50
Levodopa equivalent dosage (mg)	NA	NA	706 ± 328

An asterisk (*) indicates a significant difference between the non-PD and PD participants (p < 0.001, Wilcoxon rank sum test). Each cell shows the mean ± s.e.m, where applicable. On/off: on-medication/off-medication. NA: not applicable.

### Task procedures

The participants were required to sit with their hands palm down on a desk and their arms uncrossed (arms-uncrossed condition) or with their arms crossed (arms-crossed condition). In the arms-crossed condition, the left arm was placed over the right arm, and the arms touched each other at the distal end of the forearm.

Solenoid skin contactors (Uchida Denshi, Tokyo, Japan) were used to deliver brief tactile stimulation (10 ms duration) to the dorsal surface of the ring finger of each hand. The distance between the ring fingers was 20 cm in all conditions. A response button was placed under the tip of each index finger. During the experiments, the participants had to close their eyes while white noise (80 dB) was played via headphones placed over the participants’ ears. Therefore, the stimulus could be neither seen nor heard; the participants could only feel the tactile stimulation of the skin.

Two successive stimuli were delivered, one to each hand, separated by one of 12 randomly assigned stimulus onset asynchronies (SOAs) from -960 to 960 ms (-960, -480, -240, -120, -60, -30, 30, 60,…, 960 ms) in the arms-uncrossed condition and from -1920 to 1920 ms (-1920, -960, -480, -240, -120, -60, 60, 120,…, 1920 ms) in the arms-crossed condition. In positive SOAs, the right hand was stimulated earlier than the left and vice versa. The participants were required to judge the order of the two stimuli and had to press a button with the index finger of the hand that had been stimulated second. To prevent the participants from making a decision from the first stimulus alone, each experiment included catch trials, in which two successive stimuli with an interval of 960 ms (arms-uncrossed condition) and 1920 ms (arms-crossed condition) were delivered to only one hand (right or left). The participants were encouraged to respond as soon as possible after the delivery of the second stimulus. When the reaction time was larger than 6000 ms or the participants responded before the second stimulus, that trial (with the same SOA) was repeated at the end of each experiment. No feedback was given to the participants about the reaction time or whether their responses were correct/incorrect.

All of the participants, except for two young participants and eight PD participants, took part in two separate experiments in the following order: with the arms uncrossed and with the arms crossed. To increase our statistical power to detect the difference in the temporal resolution in the arms-uncrossed condition, we started from the arms-uncrossed condition in all of the participants, thereby maximizing the number of participants with valid data in the arms-uncrossed condition. The two young participants and eight PD participants were not able to participate in the second experiment (arms-crossed condition) owing to fatigue. Each experiment consisted of eight epochs, in which 12 test trials with different SOAs and 2 catch trials were permuted in a random order. Thus, one experiment consisted of 112 trials including 16 catch trials. Inter—trial intervals were randomly chosen within the range of 1.5 to 2.5 s.

The participants took part in a single stimulus task before each TOJ experiment with the arms crossed or uncrossed. A single stimulus was delivered to only one of the two hands in a random order for 20 trials, and the participants were required to react by pressing a button with the index finger of the stimulated hand. This task was necessary because we had to evaluate how many of the errors in the TOJ task resulted from the mere inability to report a correct hand when a single stimulus was delivered to a single hand. The single stimulus task also served as a period during which participants became familiar with the response buttons and tactile stimuli.

### Data analysis

The response data were sorted by stimulation interval to calculate the order-judgment probabilities that the right hand was stimulated earlier (or the left hand was stimulated later) in the uncrossed (*p*_*u*_) and crossed (*p*_*c*_) conditions. The data from trials in which the reaction time was greater than the 75th percentile by 1.5 times the interquartile range (in the arms-uncrossed condition) or longer than 6000 ms were excluded as outliers. The order-judgment probability in the uncrossed condition (*p*_*u*_) was fitted by a cumulative density function of a Gaussian distribution as follows [13]:

$$p_{u}(t)=(p_{\text{max}}-p_{\text{min}})\int_{-\infty }^{t}\frac{1}{\sqrt{2\pi }\sigma_{u}}\text{exp}\left(\frac{-{\left(\tau -d_{u}\right)}^{2}}{2\sigma_{u}^{2}}\right)d\tau +p_{\text{min}}$$ (1)

where *t*, *d*_*u*_, *σ*_*u*,_ *p*_*max*_, and *p*_*min*_ denote the SOA, size of the horizontal transition, temporal resolution, and upper and lower asymptotes of the judgment probability, respectively. The temporal resolution (*σ*_*u*_) corresponds to the SOAs that yielded 84% correct responses (relative to the asymptotes).

The order-judgment probability (right hand stimulated first) in the arms-crossed condition (*p*_*c*_) was assumed to be flipped from the order-judgment probability in the uncrossed condition (*p*_*u*_) in a manner formulated as follows:

$$p_{c}(t)=f_{l}(t)\left\{1-p_{u}(t)\right\}+\left\{1-f_{r}(t)\right\}p_{u}(t),$$ (2)

$$f_{l}(t)=A_{l}\cdot \text{exp}\left(\frac{-{\left(t-d\right)}^{2}}{2\sigma_{f}{}^{2}}\right)+c,$$ (3)

$$f_{r}(t)=A_{r}\cdot \text{exp}\left(\frac{-{\left(t-d\right)}^{2}}{2\sigma_{f}{}^{2}}\right)+c$$ (4)

where *f*_*l*_ denotes the flip probability of judgment from ‘left first’ to ‘right first’ and *f*_*r*_ denotes the flip probability of judgment from ‘right first’ to ‘left first’. We estimated five parameters in the flip probabilities that follow the Gaussian functions shown in Equations 3 and 4: the peak flip amplitude of the Gaussian functions (*A*_*l*_ and *A*_*r*_), the size of the horizontal transition (*d*), the time window of the flip (*σ*_*f*_), and a constant error rate (*c*). The constant error rate (*c*) corresponds to the degree of general error in each response, and the peak flip amplitudes of the Gaussian functions (*A*_*l*_ and *A*_*r*_) show the tendency of judgment reversal at short SOAs that subside at longer intervals. Moreover, we calculated the net peak flip amplitudes (*Ã*_*l*_ and *Ã*_*r*_) as follows [29]:

$${\tilde{A}}_{l}=\text{max}\left(A_{l}\cdot \text{exp}\left(\frac{-{\left(t-d\right)}^{2}}{2\sigma_{f}{}^{2}}\right)\cdot (1-p_{u}(t))\right),\text{and}$$ (5)

$${\tilde{A}}_{r}=\text{max}\left(A_{r}\cdot \text{exp}\left(\frac{-{\left(t-d\right)}^{2}}{2\sigma_{f}{}^{2}}\right).\;p_{u}\left(t\right)\right).$$ (6)

Matlab was used with the optimization toolbox (MathWorks, Natick, MA, USA) for fitting while minimizing the Pearson chi-squared statistic, which reflects the discrepancy between the sampled order-judgment probability and the prediction using the four-parameter model.

The median of each estimated parameter was compared across the three participant groups (young, non-PD elderly, and PD elderly participants) in each condition (arms-uncrossed and crossed) by using the Wilcoxon rank-sum test (exact test). The level of statistical significance was set to 0.05/3 after a Bonferroni correction for multiple comparisons. A non-parametric test was used because 13 out of 21 distributions significantly deviated from normal over all 7 parameters (Lilliefors test): the temporal resolution (*σ*_*u*_, young: *p* = 0.012), the net peak flip amplitude (*Ã*_*l*_, PD: *p* = 0.036; *Ã*_*r*_, young: *p* = 0.0092), the constant error rate (*c*, young: *p* < 10^-10^, non-PD: *p* = 0.0071), the time window of the flip (*σ*_*f*_, non-PD: *p* = 0.0024, PD: *p* = 0.018), the error rate in catch trials (young: *p* < 10^-10^, non-PD: *p* = 0.0026, PD: *p* = 0.016), and the error rate in single stimulus trials (young: *p* < 10^-10^, non-PD: *p* < 0.0001, PD: *p* < 0.0001). Accordingly, we used Spearman’s rank correlation coefficient rather than Pearson’s when correlations between two parameters were calculated. The 95% bootstrap confidence interval of the mean was also calculated for each estimated parameter.

To evaluate the effect size in each comparison between two groups, we calculated the Mann-Whitney test statistic “*U*” divided by the product of the number of participants in each group:

$$\frac{U}{n_{1}\times n_{2}}=\frac{ranksum_{2}-n_{2}(n_{2}+1)/2}{n_{1}\times n_{2}}$$ (7)

where *n*_*1*_ and *n*_*2*_ represent the number of participants in groups 1 and 2, and *ranksum*_2_ represents the sum of the ranks of members in group 2 when the members of groups 1 and 2 are ordered from smallest to largest. The statistic (*U*/(*n*_*1*_×*n*_*2*_)) reflects the probability of a sample from group 2 being larger than a sample from group 1 when each sample is randomly selected from each group. Thus, *U*/(*n*_*1*_×*n*_*2*_) computes to 1) the value of 0 when all data in group 2 are smaller than those in group 1, 2) the value of 0.5 when the distributions of the two groups are identical, and 3) the value of 1 when all data in group 2 are greater than in group 1.

A three-way mixed model ANOVA was used to compare mean reaction time with a between-subject main factor (the participant group: young, non-PD elderly, and PD elderly participants), and two within-subject main factors (the posture: uncrossed, and crossed; the SOA: -960, -480, -240, -120, -60, 60,…, and 960 ms), and their interactions. Post-hoc tests of simple main effects, and/or simple, simple main effects were performed for each of significant interactions. Significance of the post-hoc tests were judged by using the Ryan’s method for multiple comparison [30].

## Results

### Arms-uncrossed condition

**Fig 1:**
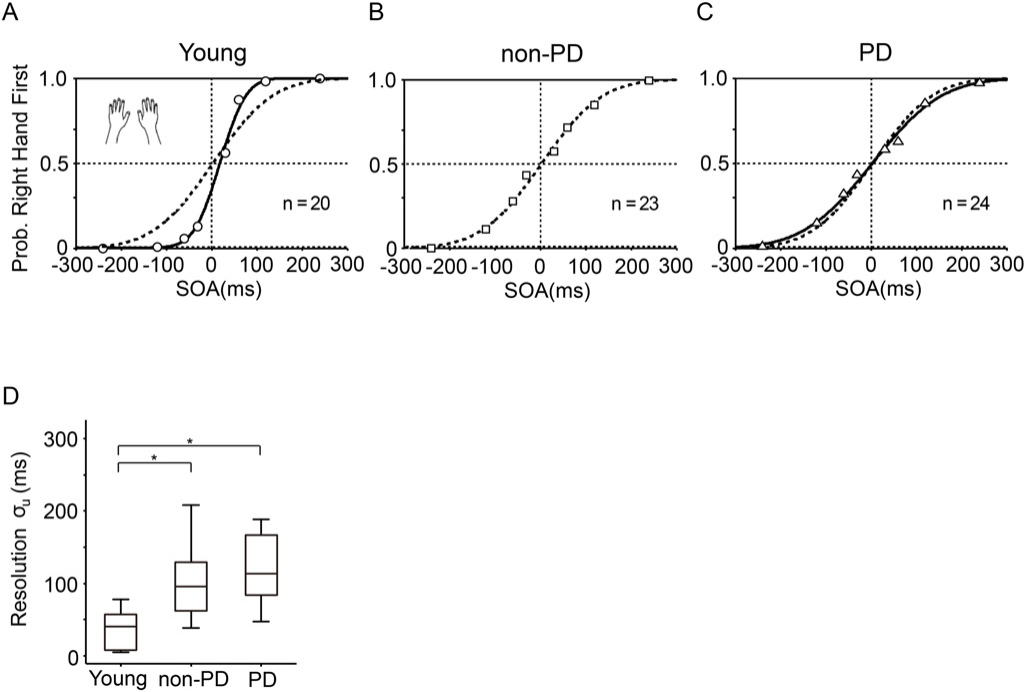
Temporal order judgment in the arms-uncrossed condition. (A-C) The order-judgment probability (ordinate) that the right hand was stimulated earlier than the left is plotted against the stimulation onset asynchrony (SOA, abscissa) for the young (A, circles), non-PD elderly (B, squares), and PD elderly participants (C, triangles). A positive SOA indicates that the right hand was stimulated first. The sigmoid curves indicate the results of the model fitting (Equation 1). A broken curve in (B) shows the result for the non-PD elderly participants and the same broken curve is superimposed in (A) and (C) for the sake of comparison with the young (A) and elderly-PD participants (C). Each symbol represents 160 (A, 8 trials×20 participants), 184 (B, 8×23), and 192 trials (C, 8×24) trials. (D) Group comparisons of temporal resolution. Each box in the box-plots show the 25, 50 (median), and 75 percentiles. The whiskers extend to the most extreme data points not considered as outliers that falls outside the range of 1.5 times of the interquartile range from either end of each box. The brackets with asterisks indicate that the median was significantly different (p < 0.05/3, Wilcoxon rank sum test after Bonferroni corrections for multiple comparisons).

With arms uncrossed, the order-judgment probability (*p*_*u*_) that the right hand was stimulated first was closely approximated by a monotonic sigmoid function (Equation 1) in the young, non-PD elderly, and PD elderly participants (Figs. 1A-C). The sigmoid curves clearly show that the temporal resolution (*σ*_*u*_, the SOA yielding approximately 84% correct responses) was generally better in the young participants (Fig. 1A, solid curve) than the elderly participants (Fig. 1B, C). Among the elderly participants, the non-PD participants (Fig. 1B, broken curve) did not perform better than the PD patients (Fig. 1C, solid curve). Wilcoxon rank sum tests confirmed these observations (Fig. 1D); the median temporal resolution in the young participants (median = 41 ms, interquartile range (IQR) = 50 ms) was significantly better than in the elderly participants with PD (median = 114 ms, IQR = 84 ms; *p* < 0.0001, *U*/(20×24) = 0.97) and without PD (median = 96 ms, IQR = 67 ms; *p* < 0.0001, *U*/(20×23) = 0.92), but the temporal resolution was not significantly different between the non-PD and PD participants (p = 0.078, *U*/(20×23) = 0.62; α = 0.05/3, Bonferroni corrected). The temporal resolution in the PD patients did not significantly correlate with disease severity (UPDRS-III severity score, Spearman’s *ρ* = 0.0039, *p* = 0.99), disease duration (*ρ* = -0.011, *p* = 0.96), medication (Levodopa equivalent doses [31], *ρ* = -0.15, *p* = 0.48), or the score of the Mini-mental state examination (*ρ* = 0.13, *p* = 0.54).

The lower bound of the sigmoid function (*P*_min_) was close to zero, and the upper bound (*P*_max_)) was close to one in all three groups (Table 2). Accordingly, the error response rates in the catch trials (double stimulation to the same hand, Fig. 1E) and in the single stimulus trails were close to zero in all groups (Fig. 1F, Table 2). The results show that the young and elderly participants seldom erred in reporting the hand that was stimulated as long as a single hand was stimulated with the arms uncrossed.

**Table 2:** Comparison of the mean model parameters.

		Young	Non-PD elderly	PD
		(n = 20)	(n = 23)	(n = 24)
Arms uncrossed	*σ*_*u*_ (ms)*	36 ± 26, [26, 47]	109 ± 58, [90, 140]	133 ± 68, [111, 166]
	*d*_*u*_ (ms)	20 ± 27	4.2 ± 41	4.2 ± 61
	*P*_*min*_	0	0.0009 ± 0.003	0.0004 ± 0.002
	*P*_*max*_	1	1	0.9996 ± 0.002
	*Single*	1	1	0.998 ± 0.011
	*Catch*	0.997 ± 0.014	1	0.997 ± 0.014
Arms crossed	*Ã*_*l*_	0.48 ± 0.28, [0.36, 0.61]	0.43 ± 0.28, [0.32, 0.55]	0.40 ± 0.25, [0.27, 0.51]
	*Ã*_*r*_	0.37 ± 0.22, [0.29, 0.49]	0.26 ± 0.20, [0.18, 0.34]	0.31 ± 0.20, [0.21, 0.40]
	*c* *	0.019 ± 0.04, [0.0050, 0.041]	0.21 ± 0.19, [0.14, 0.29]	0.23 ± 0.17, [0.15, 0.31]
	*σ*_*f*_ (ms) *	278 ± 130, [229, 349]	651 ± 519, [477, 901]	894 ± 1081, [544, 1758]
	*d* (ms)	–23 ± 131	-85 ± 363	19.2 ± 334
	*Single* *	0.02 ± 0.030, [0.0035, 0.028]	0.16 ± 0.21, [0.16, 0.39]	0.17 ± 0.20, [0.12, 0.32]
	*Catch* *	0.01 ± 0.030, [0.0083, 0.036]	0.26 ± 0.29, [0.094, 0.27]	0.18 ± 0.19, [0.094, 0.29]

See Equations 1–6 for the definition of each parameter. Numbers in brackets show the 95% bootstrap confidence intervals of the mean with 10000 bootstrap samples. Each asterisk indicates that there was a significant difference between the young and non-PD elderly participants (p < 0.001, Wilcoxon rank sum test). Note that no parameter was significantly different between the PD and non-PD participants.

### Arms crossed condition

**Fig 2:**
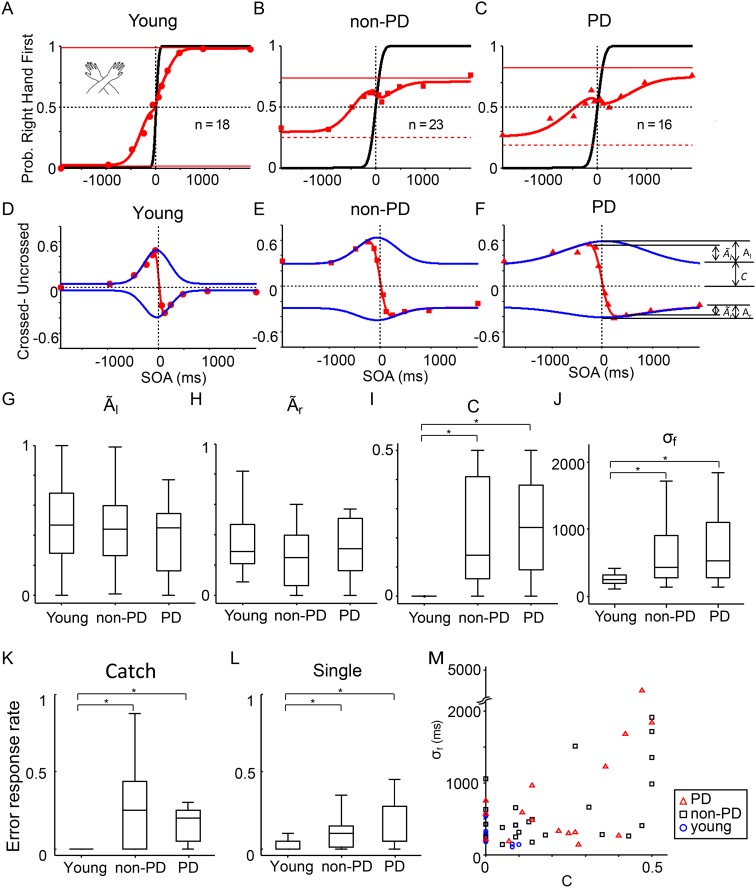
Temporal order judgment in the arms-crossed condition. (A-C) The red symbols indicate the probability of a right-hand first judgment in the arms-crossed condition plotted against the SOA for the young (A), non-PD elderly (B), and PD elderly participants (C). The black and red curves indicate the results of the model fitting in the uncrossed (Equation 1) and crossed (Equation 2–4) conditions. Each symbol represents 144 (A, 8×18), 184 (B, 8×23) and 128 (C, 8×16) trials. The other conventions are as in Fig. 1A. (D-F) The difference between the order-judgment probability in the crossed and uncrossed condition (ordinate) are plotted against the stimulation onset asynchrony (SOA, abscissa). The difference shown in (D), (E), and (F) was calculated using data from the young (A), non-PD elderly (B), and PD elderly participants, respectively. The upward and downward Gaussian curves (blue) correspond to the flip functions, *f*_l_ and *f*_r_, of the judgment probabilities as defined in Equations (3) and (4). The net peak flip amplitudes (*Ã*_*l*_ and *Ã*_*r*_ in Equations (5) and (6)) and the probability of generic error (*c* in Equations (3) and (4)) are indicated in (F). (G-L) Group comparisons: the net left-to-right flip probabilities (*Ã*_*l*_) (G), those of the reverse (*Ã*_*r*_) (H), the constant error rate (*c*) (I), the time window of the flip (*σ*_*f*_) (J), and error response rates in the catch (K) and the single stimulus trials (L). The box-plot conventions are as Figs. 1D-F. The brackets with asterisks show that the median was significantly different (p < 0.01, Wilcoxon rank sum test after the Bonferroni correction for multiple comparisons). (M) The time window of the flip (*σ*_*f*_, ordinate) plotted against the constant error rate (*c*, abscissa). Data from the young (circles), non-PD elderly (squares), and PD elderly participants (triangles) are shown with different symbols. Note a significant correlation between the two parameters (Spearman’s *ρ* = 0.36, *p* = 0.0055).

With arms crossed, both the young (red circles, Fig. 2A) and elderly participants (red squares and triangles, Figs. 2B, C) reported inverted judgment more often than in the uncrossed condition (sigmoid curves in Fig. 2A-C), which is in agreement with previous studies [13, 18, 29]. The difference between the order-judgment probability in the arms-crossed condition and the arms-uncrossed condition (red symbols in Figs. 2D-F) was biased by the constant error rate (*c*) and had positive (*Ã*_*l*_) and negative peaks (*Ã*_*r*_) that was approximated using two Gaussian functions with different peaks (*A*_*l*_ and *A*_*r*_ in Equations 3 and 4) and a single standard deviation (*σ*_*f*_). The net peak flip probabilities (*Ã*_*l*_ and *Ã*_*r*_, insets in Fig. 2F) represent the maximum probability of judgment reversal from “right-hand first” to “left-hand first” (*Ã*_*r*_) and from “left-hand first” to “right-hand first” (*Ã*_*l*_), which were added to the baseline error rate *c* (Fig. 2F, inset). When the model parameters were calculated for each participant, the median of the net peak flip probabilities were not significantly different among the three groups (*Ã*_*l*_ and *Ã*_*r*_, Figs. 2G, H). By contrast, the median of the constant error rate (*c*: the degree of general error in each response) was significantly greater in the elderly participants (non-PD: median = 0.14, IQR = 0.35; PD: median = 0.24, IQR = 0.28) than in the young participants (Fig. 2I, median = 0, IQR = 0; *p* < 0.0001, *U*/(18 × 23) = 0.86 for young/ non-PD; *p* < 0.0001, *U*/(18 × 16) = 0.87 for young/ PD; Wilcoxon rank sum tests, *α* = 0.05/3, Bonferroni corrected). The median of the time window of the flip (*σ*_*f*_ in Equations 3 and 4), which reflected the time window of the judgment reversal, was also significantly greater in the elderly participants (non-PD: median = 430 ms, IQR = 630 ms, *p* = 0.00014, *U*/(18 × 23) = 0.77; PD: median = 530 ms, IQR = 820 ms, *p* = 0.0035, *U*/(18 × 16) = 0.77; *α* = 0.05/3) than in the young participants (median = 240 ms, IQR = 130 ms).

Accordingly, the medians of the error rate in the catch trials (Fig. 2K) and the single stimulus trials (Fig. 2L) were also significantly worse in the elderly participants than in the young participants (catch: *p* = 0.00094, *U*/(18 × 23) = 0.75 for young/ non-PD, *p* < 0.0001, *U*/(18 × 16) = 0.84 for young/ PD; single: *p* = 0.00035, *U*/(18 × 23) = 0.79 for young/ non-PD, *p* = 0.00019, *U*/(18 × 16) = 0.82 for young/ PD). In addition, the constant error rate was significantly correlated with the time window of the flip (Spearman’s *ρ* = 0.36, *p* = 0.0055, *t*(55) = 2.9; Fig. 2M) and the error rate in the catch trials (*ρ* = 0.53, *p* < 0.0001, *t*(55) = 4.6; not shown) and in the single stimulus trials (*ρ* = 0.43, *p* = 0.00077, *t*(55) = 3.6; not shown). The results show that the elderly participants with or without PD generally erred more in reporting which hand was stimulated, even when a single hand was stimulated.

In addition, it is worth noting that there was not a single parameter that differed significantly between the non-PD and PD elderly participants. The 95% confidence intervals of the mean from the two groups overlapped (Table 2). The effect size, *U*/(*23*×*16*), was mostly close to 0.5: 0.49 (*Ã*_*l*_), 0.55 (*Ã*_*r*_), 0.53 (*c*), 0.52 (*σ*_*f*_), 0.45 (error rate in catch trials), and 0.49 (error rate in single-stimulus trials). The number of participants required to make these effects significant are 5000 (*Ã*_*l*_), 280 (*Ã*_*r*_), 1000 (*c*), 1600 (*σ*_*f*_), 300 (error rate in catch trials), and 6100 (error rate in single-stimulus trials) participants for each group. These results clearly show that these small effects would have never reached significance unless we recruited hundreds of participants.

### Reaction time

**Fig 3:**
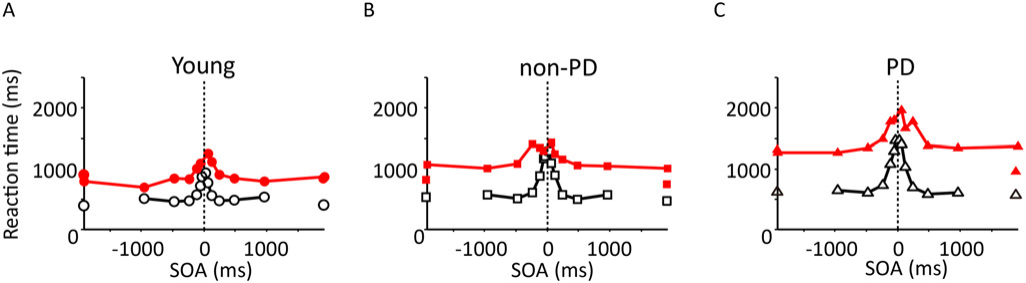
Comparisons of reaction time. The mean reaction time (ordinate) is plotted against the SOA (abscissa) for the young (A), non-PD (B), and PD participants (C). The colors differentiate between the arms-crossed (red) and -uncrossed (black) conditions.

Reaction time was generally shorter in the young participants (Fig. 3A) than in the elderly participants (Figs. 3B, C). In addition, reaction time was generally longer in the PD patients compared with the non-PD elderly participants (Figs. 3B, C). A three-way mixed model ANOVA (group × posture × SOA; group: between-subject factor; posture and SOA: within-subject factors) revealed that the three main effects, group (young, non-PD, PD; *F*(2, 54) = 18.8, *p* < 0.0001), posture (uncrossed, crossed; *F*(1, 54) = 121, p < 0.05), and SOA (-960, -480, …, 960; *F*(9, 486) = 51.4, *p* < 0.0001), and all interactions (group × posture: *F*(2, 54) = 6.9, *p* < 0.0022; group × SOA: *F*(18, 486) = 4.2, *p* < 0.0001; posture × SOA: *F*(9, 486) = 5.0, *p* < 0.0001; group × posture × SOA: *F*(18, 486) = 2.8, *p* = 0.0001) were significant. Post-hoc tests for the simple main effect of group showed on average that the mean reaction time was significantly different between any combination of the three groups (young/ PD: *p* < 0.0001, *t*(32) = 5.9; non-PD/ PD: *p* = 0.0022, *t*(38) = 3.2; young/ non-PD: *p* = 0.0034, *t*(40) = 3.1, Ryan’s method; young: 720 ± 20 ms, non-PD: 990 ± 22 ms, PD: 1300 ± 31 ms; mean ± s.e.m.). However, it is worth noting that the three-way interaction qualified the main effect in many ways. For example, the simple, simple main effects of group did not reach a level of significance at longer SOAs (-960, -480, -240, 480, 960 ms) when the arms were uncrossed, but were significant at any SOAs when the arms were crossed. That is, the group difference was more marked when the arms were crossed (Fig. 3, red circles) than when the arms were uncrossed (black squares). Longer reaction time in the PD patients compared with the non-PD participants, ~300 ms greater on average, indicates that the motor disorder due to Parkinsonism was present even with medication.

## Discussion

In the present study, we examined tactile temporal order judgment in young adults, healthy elderly participants and elderly participants with PD. We found clear effects of aging in both conditions (arms uncrossed and crossed). With arms uncrossed, the temporal resolution was significantly worse in the elderly participants compared with the young participants. With arms crossed, judgment reversals were observed at relatively short SOAs (i.e., < 500 ms) in the young participants but over the entire range (up to ~2 s) in the elderly participants. By contrast, we were not able find any significant differences between the non-PD and PD participants, except that reaction times were significantly longer in the PD patients.

### Effects of aging on temporal resolution

The temporal resolution in the elderly participants was worse than in the young participants when the participants’ arms were uncrossed. These results agree with previous studies [23, 24]. The age-related decline in tactile acuity, reported here and in previous studies, may be attributed to a general slowing of nerve conduction, not only in the first-order sensory nerves [32] but also in the callosal fibers used for interhemispheric transfer [20]. It is worth noting that a slowing of interhemispheric nerve conduction not only results in a delay but also leads to an increase in the variability of the delay [20]. This increase in variability should directly affect any temporal processing, including TOJ, SJ and temporal discrimination tasks. It is of course possible that general cortical atrophy in normal aging [33] underlies the age-related decline in temporal resolution.

### Effects of aging on judgment errors in the arms-crossed condition

In the arms-crossed condition, judgment reversal was observed in both the young and elderly participants, as previously reported in non-elderly participants. Among the parameters of the model fitting, the net peak flip probabilities (*Ã*_*l*_, *Ã*_*r*_) of the elderly participants were not significantly different from the young participants. This new finding indicates that the degree of judgment reversal due to arm-crossing per se was comparable in both age groups with short SOAs.

However, the median time window of the flip (*σ*_*f*_), which reflects the time window of the judgment reversal, was significantly larger in the elderly participants (non-PD: 430 ms, PD: 530 ms) than in the young participants (240 ms, Fig. 2J). In addition, the constant error rate in TOJ (Fig. 2I), which reflects the general error rate in choosing which hand, and the error rates in the catch (Fig. 2K) and single stimulus trials (Fig. 2L) were significantly larger in the elderly participants than in the young participants whose median error rates were zero. That is, the elderly participants generally made more errors not only at SOAs as long as ~2 s but also when a single stimulus was delivered to a single hand. In addition, the 4 parameters, the time window of the flip, the constant error rate and error rates in catch and single stimulus trials generally correlated with each other. Thus, the increase in the 4 error parameters in the elderly participants is likely to reflect the same effect of aging.

It is now widely accepted that the brain initially maps a stimulus that was delivered to one of the crossed hands (e.g., the left hand located to the right of the right hand) to the wrong hand (the right hand to the left of the left hand), then remaps the stimulus to the correct hand (the left hand) within 0.3–0.4 s (or even faster) after the stimulus delivery [34–39] in non-elderly participants. This idea originated from observations of curved saccades that occur when participants are required to make a speeded saccade to a hand that is touched while their arms are crossed [40, 41]. When the saccade latency is less than 200 ms, the eyes often start to move toward the wrong hand and then change their direction toward the correct hand, resulting in a curved trajectory. The probability of performing a curved saccade drops to less than 5% when the saccade onset latency is larger than 250 ms, but it increases up to 70–80% when the onset latency is as short as 160 ms [41, 42]. It should be noted that the participants were young in these studies (20–37 years old in Overvliet, Azanon and Soto-Faraco [41] and 21–34 years old in Nakano, Neshime, Shojima and Kitazawa [42]). The increase in the probability of curved saccades up to 80% at shorter onset latencies indicates that the initial mapping to the wrong hand is a reflexive process shared among young participants, and the corrective remapping follows within a very short period. Based on the present results, we speculate that elderly participants share the initial incorrect mapping process with young participants but have deficits in the second remapping process.

### Lack of contribution of the basal ganglia to TOJ

Consistent with Nelson, Premji, Rai, Hoque, Tommerdahl and Chen [12], the temporal resolution of the PD patients was comparable with the non-PD participants. Judgment errors in the arms-crossed condition were similarly observed in both the non-PD and PD participants. The lack of significant differences between the non-PD and PD participants suggests that the age-related changes in TOJ across hands do not result from changes in the basal ganglia or the dopaminergic system in either the arms-uncrossed condition or the arms-crossed condition.

However, the present results must be evaluated with caution because the participants with PD joined the experiments during medicated periods. It is possible that medication improved the temporal resolution and obscured any significant effects of the PD condition that would have been detected if they had been tested while unmedicated. However, we believe that this is unlikely due to the following reasoning. First, reaction times were significantly longer in the PD patients than in the age-matched controls by 200–300 ms on average (Fig. 3). In addition, the mean UPDRS motor score was as large as 13.8 (s.e.m = 6.8) even under medication. These results suggest that typical motor deficits still remained in spite of the medications. Second, three patients who were tested during their off-medication period showed no improvement in temporal resolution compared with their on-medication tests. This agrees with Nelson, Premji, Rai, Hoque, Tommerdahl and Chen [12] who also reported no improvement in temporal resolution during on-medication periods compared with off-medication periods.

Thus, it may be questioned why PD patients have deficits in temporal discrimination (TD) and SJ tasks [6–8] but not in the TOJ tasks in the present and previous studies [12]. If SJ is a prerequisite to TOJ, deficits in SJ should lead to impairments in TOJ. However, these findings are not surprising if TOJ is distinct from TD and SJ. Indeed, it is generally recognized that TOJ and SJ involve different processes [13, 14, 16, 43]. For example, Jaśkowski [16] explained the results from a ternary-response paradigm (choice from “A then B”, “B then A”, or “simultaneous”) by assuming that there are two parallel mechanisms, one for SJ and another for TOJ. We further speculate that TD and SJ are variants of an interval discrimination task in which a zero interval and a non-zero interval are discriminated. In interval discrimination, participants must pay attention to the “length” of time demarcated by two events, whereas they do not need to measure this length in TOJ tasks. Therefore, we might be able to explain the previous and present findings as follows: interval discrimination tasks, which require a measurement of the “length” of time, are generally impaired by PD [10, 11], but TOJ, which does not require a measurement of the time length, is not impaired.

The lack of critical contribution of the basal ganglia to TOJ is also supported by recent neuroimaging studies. Takahashi, Kansaku, Wada, Shibuya and Kitazawa [17] examined brain activity during the same TOJ task used in the present study and showed that several cortical areas over the prefrontal, parietal, and temporal cortices were activated. The caudate nucleus was also activated, but the activation was less significant than the activation in the cortical areas. Davis, Christie and Rorden [44] reported that the temporal parietal junction was specifically engaged in the visual TOJ task but did not show any involvement of the basal ganglia. Although the sensory modalities were different in these two studies, both reported major activations in the same bilateral temporal and parietal regions. The cortical regions shared across sensory modalities are likely to play central roles in determining the temporal resolution of TOJ, which is known to be similar across sensory modalities [45, 46]. By contrast, it is unlikely that the basal ganglia are a crucial component for TOJ in general.

Finally, we discuss why two studies, our study and Nelson, Premji, Rai, Hoque, Tommerdahl and Chen [12], reported no effects of PD on TOJ, whereas Fiorio, Valente, Gambarin, Bentivoglio, Ialongo, Albanese, Barone, Pellecchia, Brancati, Moretto, Fiaschi and Tinazzi [7] reported clear impairments in temporal resolution. Two differences might explain this discrepancy. First, Fiorio, Valente, Gambarin, Bentivoglio, Ialongo, Albanese, Barone, Pellecchia, Brancati, Moretto, Fiaschi and Tinazzi [7] required participants to judge not only temporal order but also simultaneity, whereas TOJ was the only task in the other two studies. Assuming that TOJ and SJ are distinct processes and that SJ is impaired by PD, the concurrent SJ task must have disturbed the TOJ task more severely in PD patients than in age-matched controls. Second, Fiorio, Valente, Gambarin, Bentivoglio, Ialongo, Albanese, Barone, Pellecchia, Brancati, Moretto, Fiaschi and Tinazzi [7] examined participants with familial PD due to a PINK1 mutation, whereas idiopathic PD patients with no clear genetic background were recruited in the other two studies. It is possible that patients with familial PD due to a PINK1 mutation have somatosensory deficits beyond the basal ganglia [47] that could have resulted in impairments in both SJ and TOJ tasks.

Taken together, we found age-related declines in temporal resolution during the arms-uncrossed condition and deficits in elderly participants when remapping tactile signals in the arms-crossed condition. By contrast, these effects were not altered by the presence of PD. Therefore, we conclude that thalamo-cortico-striatal circuits and the dopaminergic system are not critical for TOJ.
